# Distortion of auditory space during visually induced self-motion in depth

**DOI:** 10.3389/fpsyg.2014.00848

**Published:** 2014-08-05

**Authors:** Wataru Teramoto, Zhenglie Cui, Shuichi Sakamoto, Jiro Gyoba

**Affiliations:** ^1^Department of Computer Science and Systems Engineering, Muroran Institute of TechnologyMuroran, Japan; ^2^Research Institute of Electrical Communication, Tohoku UniversitySendai, Japan; ^3^Department of Psychology, Graduate School of Arts and Letters, Tohoku UniversitySendai, Japan

**Keywords:** veciton, auditory localization, visual-vestibular interaction, space perception, self-motion perception

## Abstract

Perception of self-motion is based on the integration of multiple sensory inputs, in particular from the vestibular and visual systems. Our previous study demonstrated that vestibular linear acceleration information distorted auditory space perception (Teramoto et al., 2012). However, it is unclear whether this phenomenon is contingent on vestibular signals or whether it can be caused by inputs from other sensory modalities involved in self-motion perception. Here, we investigated whether visual linear self-motion information can also alter auditory space perception. Large-field visual motion was presented to induce self-motion perception with constant accelerations (Experiment 1) and a constant velocity (Experiment 2) either in a forward or backward direction. During participants' experience of self-motion, a short noise burst was delivered from one of the loudspeakers aligned parallel to the motion direction along a wall to the left of the listener. Participants indicated from which direction the sound was presented, forward or backward, relative to their coronal (i.e., frontal) plane. Results showed that the sound position aligned with the subjective coronal plane (SCP) was significantly displaced in the direction of self-motion, especially in the backward self-motion condition as compared with a no motion condition. These results suggest that self-motion information, irrespective of its origin, is crucial for auditory space perception.

## Introduction

When we move in an environment, auditory input to our ears dynamically changes. This likely interferes with accurate sound localization because cues for auditory localization are primarily coordinated and centered on the head. Nevertheless, in reality, we perceive a stable auditory space^1^. This requires that the brain interpret auditory signals in reference to signals about head and body movements. Neurophysiological and psychological findings suggest that the vestibular system is crucial for providing such movement information. For example, when blindfolded listeners were rotated around a vertical axis, they perceived a physically stationary sound as displaced in a direction opposite to their self-rotation. This is known as the “audiogyral illusion” (Münsterberg and Pierce, 1894; Clark and Graybiel, 1949; Arnoult, 1950; Lester and Morant, 1970). Several recent reports showed that the direction of displacement was reversed (i.e., in the direction of vestibular stimulation) when listeners were exposed to semicircular stimulation that was too weak to induce an illusory kinesthetic sense (i.e., explicit postural and movement information) (Lewald and Karnath, 2000, 2001; see also van Barneveld and John Van Opstal, 2006). Rapid head turns can also lead to the distortion of auditory space in the perisaccadic interval, just like visual localization during or immediately before saccadic eye movements (Cooper et al., 2008; Leung et al., 2008). In addition to the information originating in the semicircular-canal system, sensory information from the macular receptors of the otolith system (utricle and saccule) also plays a role in this respect. Graybiel and Niven (1951) used a centrifuge (a slowly rotating room) to show that the perceived direction of a sound source shifted in the direction of the resultant linear gravitoinertial force (see also DiZio et al., 2001; Lackner and DiZio, 2010). Body tilts, or changes in body position relative to gravity, also systematically affect auditory localization (Teuber and Liebert, 1956; Lackner, 1973; Lewald and Karnath, 2002). Although the direction of displacement is debatable as is the effect of rotary acceleration on auditory space perception mentioned above, these studies suggest that information from the otoliths as well as the semicircular canals influence auditory localization/lateralization in azimuth. Several studies have argued that auditory mislocalization during vestibular rotary and gravitoinertial force stimulation is associated with shifts in subjective body positions or egocentric reference frames (Münsterberg and Pierce, 1894; Clark and Graybiel, 1949; Arnoult, 1950; Lester and Morant, 1970).

Furthermore, our recent study demonstrated that in addition to auditory localization/lateralization in azimuth, auditory localization in depth can also be affected by otolith signals, despite differences in cues useful for auditory localization in depth vs. azimuth (Teramoto et al., 2012). In this study, a robotic wheelchair was used to produce naturalistic linear accelerations (forward/backward). An array of loudspeakers was set along the wall to the right of participants, parallel to the motion path. A target sound was delivered from one of the loudspeakers during self-motion and blindfolded participants were asked to indicate in which direction it was perceived (a two-alternative forced choice in Experiments 1 and 2, or a pointing task in Experiment 3). The results showed that the sound position aligned with the subjective coronal plane (SCP) (or frontal plane) was displaced in the direction of self-motion. In other words, sound sources located in the traveling direction were perceived as closer to the participant than their actual locations (i.e., displacement in the direction opposite to their self-motion). More interestingly, this effect only occurred for forward motion. Thus, these studies suggest that vestibular signals, irrespective of semicircular canals or otoliths, contribute to the construction of auditory space during self-motion.

Aside from information originating in the vestibular system, the visual system could also play an important role in self-motion perception. Large-field visual motion can induce the sensation of self-motion (vection). Various properties of self-motion such as speed, distance, and heading direction can be detected from visual information (e.g., Lappe et al., 1999; Sun et al., 2004). The visual self-motion information can alter other visual perception such as object motion (e.g., Probst et al., 1984), temporal order of visual events (Teramoto et al., 2004), and depth (Watanabe et al., 2004). In the auditory localization literature, previous studies reported that rotation of a visual environment around the vertical axis caused displacement of a sound source in the direction of visual motion (i.e., in the opposite direction of induced self-motion) (Thurlow and Kerr, 1970; Cullen et al., 1992; Otake et al., 2006; see also Väljamäe, 2009 for a review). This is nearly in line with the results from the studies addressing semicircular stimulation.

The present study investigated whether visual stimulation that simulates linear self-motion affects sound localization in depth. Our previous study demonstrated that vestibular linear stimulation caused the perceived displacement of sound sources in the opposite direction of self-motion. However, it is not clear whether this is the case for visual linear self-motion information. It is possible that a difference in the origin of self-motion information could produce different results. Furthermore, one advantage of visual over vestibular stimulation is that all cues for sound localization are identical between the static and visual stimulation conditions. For physical movements used in our previous study, these cues varied during the 30-ms target sound presentation between the self-motion vs. static conditions. For example, it is well known that HRTF parallax can be useful for localizing sound sources within 1 m of a listener (Zahorik et al., 2005). The HRTF parallax was changed by a maximum of 5 mm during the sound presentation. This was a very small change compared to the accuracy of distance perception based on the HRTF parallax (Kim et al., 2001), but might contribute to sound localization during self-motion. Thus, the present study can reveal which is more critical for the distortion of auditory space during self-motion: the continuous change in acoustic cues or self-motion information itself.

## Experiment 1

### Methods

#### Participants

Twelve participants (three females, nine males; age range: 21–40 years), including two of the authors, participated in Experiment 1. All participants except the authors were naïve to the purpose of the experiment. All participants had normal or corrected-to-normal vision, normal hearing, and no vestibular dysfunction. Informed consent was obtained from each participant before the experiment. The procedure was approved by the Ethics Committee of the Research Institute of Electrical Communication of Tohoku University. These criteria also apply to Experiment 2.

#### Apparatus

**Figure 1:**
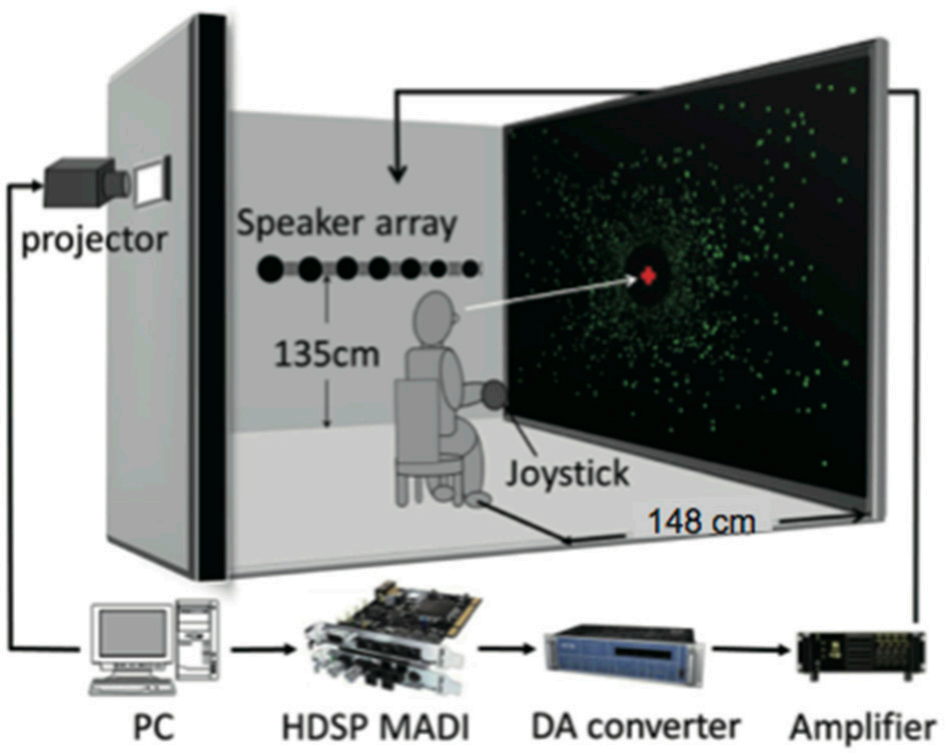
**Schematic diagram of experimental setup**. Large-field visual motion was presented to induce self-motion perception in either a forward or a backward direction. During participants' experience of self-motion, a short noise burst was delivered from one of the loudspeakers aligned parallel to the motion direction along a wall to the left of the listener.

The experiment was conducted in a sound attenuated room in the Research Institute of Electrical Communication, Tohoku University. The maximum sound pressure level of ambient environmental noise in the room was 27 dB (A-weighted). Figure 1 shows a diagram of the experimental setting. The experiment was controlled by an IBM-compatible personal computer (Dell, Precision T3500). All visual stimuli were projected with a projector (SANYO, PDG-DHT100JL; refresh rate: 60 Hz; resolution: 1280 × 1024) on a 150-inch projection screen made from materials for acoustic penetration (Stewart Filmscreen, FireHawk G3). Participants were seated in a chair with a headrest, which was located 1.48 m from the center of the screen (field of view: 90 × 78°), and were asked to hold their head against the headrest. The headrest was made of wireframe parts so as not to block acoustic signals from reaching to the ears. The participants held a gamepad for making responses in their hands. An array of 11 full-range loudspeakers (HOSIDEN, 0254-7N101, 30 mm) was placed perpendicular to the screen (i.e., parallel to the anterior-posterior axis of the human body) along the wall to the left of the participants at a height of 1.35 m (almost equivalent to the height of the seated participant's ears). The central speaker of the array was placed 1.48 m lateral to the participants on their physical coronal plane. This speaker was defined as the null point (0°). The angles between the remaining speakers and the center of participants' head were 4, 8, 16, 24, and ±32°. Negative and positive values indicate the rear and frontal space, respectively. Audio data were output through audio interfaces (RME, HDSP MADI; RME, M32-DA) using a power amplifier (Mishima Planning, MP-3016).

#### Stimuli and procedure

A random-dot pattern simulating either forward or backward linear self-motion or no motion was displayed on the screen as the visual stimulus. The pattern consisted of 0.1 × 0.1° green dots (28.6 cd/m^2^) on a black background with a dot density of 15%. A red fixation circle (1.5° radius) was also displayed. Auditory stimuli consisted of 30 ms of pink noise modulated by 5-ms raised-cosine onset and offset windows at an average sound pressure level (A-weighted) of 54 dB (sampling frequency: 44.1 kHz). Pink noise was used because the spectrum of pink noise is closer than white noise in terms of what we hear in the natural world and, thus, pink noise would likely be relevant to our sound localization task.

There were two directions (forward and backward) of simulated self-motion and two accelerations (0.15 and 0.3 m/s^2^) for each direction. The reason that visual motion stimuli at a constant acceleration were used was for comparison with our previous study using vestibular stimulation (Teramoto et al., 2012). The two self-motion directions were tested in different blocks. In each block, the no motion session, where the static dot pattern was presented, was conducted first, followed by the two acceleration sessions (0.15 and 0.3 m/s^2^). The order of acceleration conditions was randomized. Two forward and two backward self-motion blocks were conducted in random order. Before these experimental blocks, one practice block without visual stimulation was conducted to check each participant's sound localization ability and to familiarize participants with the experimental procedure. Each experimental session consisted of a number of trials for the staircase procedure (described below).

At the beginning of each trial, the static random-dot pattern was presented with a fixation point. When the participants pressed a button of the gamepad, the trial was started. In the no motion condition, a target sound was presented 1 s after the participants' button press, and participants made a response. In the forward and backward conditions, after the button press, the random-dot pattern started to move. The initial velocity of simulated self-motion was 0.4 m/s (constant). As soon as participants reported self-motion perception, the velocity of simulated self-motion increased at a constant acceleration of either 0.15 or 0.3 m/s^2^. A target sound was presented when the velocity of simulated self-motion reached 1.5 m/s (i.e., 6.0 and 3.0 s after the acceleration in the 0.15 and 0.3 m/s^2^ acceleration conditions, respectively). The reason that we first presented the visual stimuli at a constant velocity was to ensure that participants sufficiently perceived self-motion when target sounds were presented. If visual stimuli at constant accelerations were presented from the beginning of each trial, target sounds could have been presented before participants perceived self-motion. One second after the presentation of the target sound, the visual stimuli disappeared and participants indicated the direction in which the sound was perceived (front or back) relative to their coronal plane. Vection onset times (VOTs) were registered by participants' pressing the button to report self-motion perception.

The test sound position varied from trial to trial according to a staircase method (Cornsweet, 1962). In one staircase sequence, the initial position of the sound was 32° (descending series), and in another staircase sequence the initial position was −32° (ascending series). These two staircase sequences were randomly intermixed in a session. Each staircase sequence was terminated after 5 reversals of the response sequence. Thus, 10 reversals were obtained in a session. Because two sessions were conducted for each self-motion condition, 20 reversals were averaged to obtain the alignment of the sound position with the SCP.

### Results and discussion

**Figure 2:**
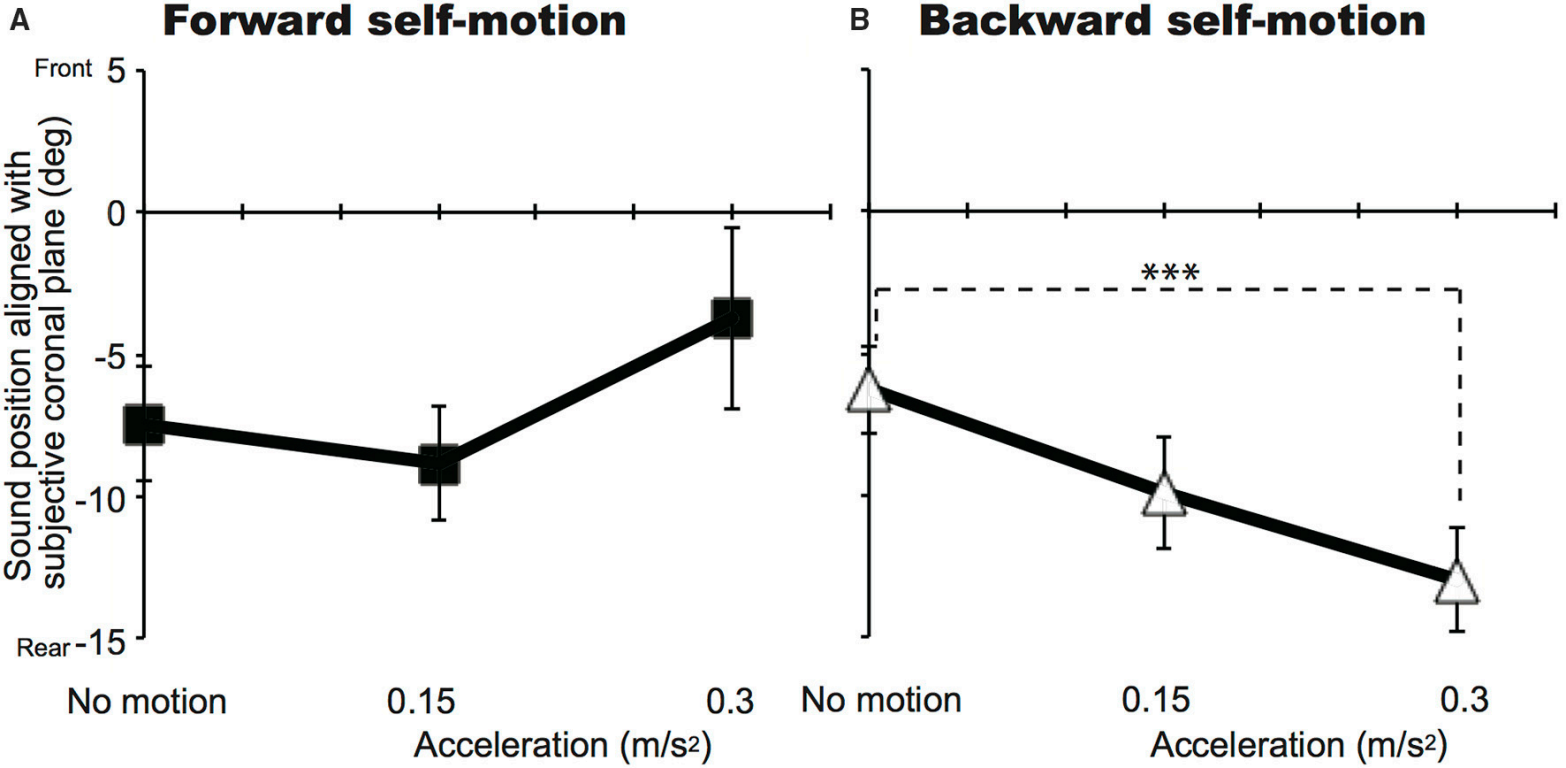
**Effects of acceleration on auditory localization in Experiment 1 for the forward (A) and backward (B) conditions**. The mean sound positions aligned with participants' subjective coronal plane are shown as a function of acceleration. The null point indicates the physical coronal plane. Error bars denote standard errors. Note that the participants experienced self-motion perception but were not physically moved. ^***^p < 0.005.

**Figure 3:**
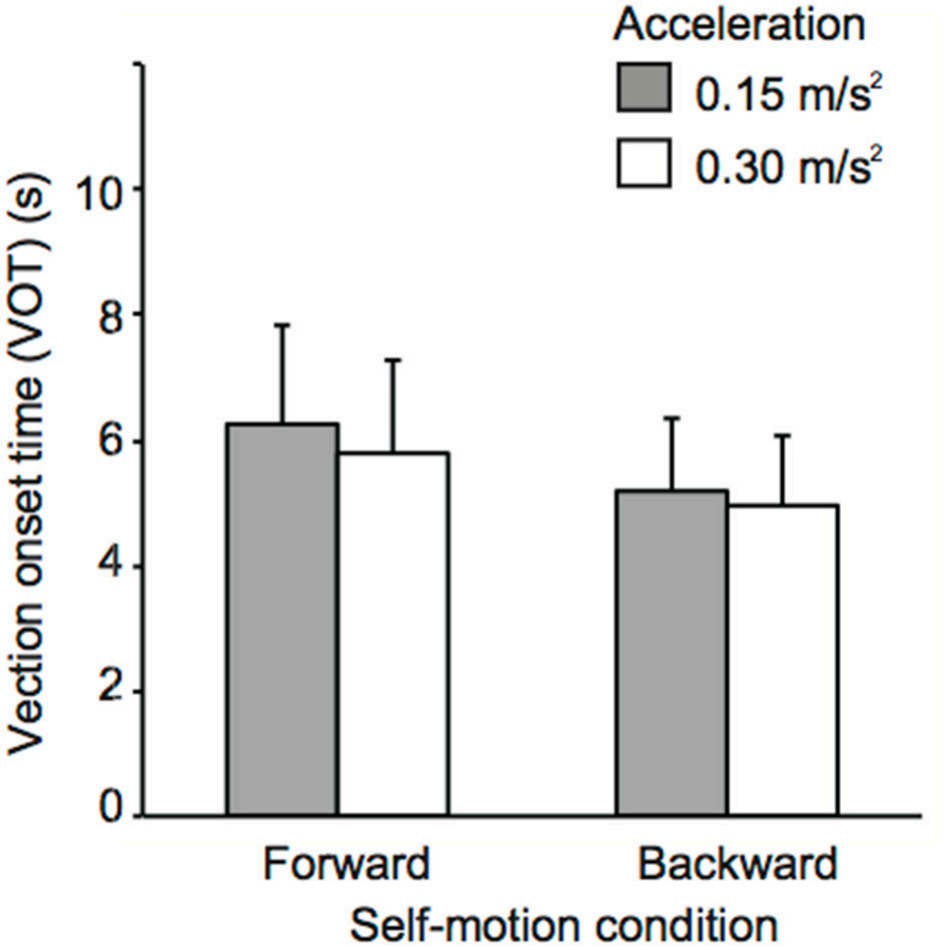
**Effects of acceleration on vection onset latency Experiment 1**. Error bars denote standard errors.

Figures 2, 3 show mean sound positions aligned with participants' SCP and mean VOTs in Experiment 1, respectively. The null point indicates a sound position aligned with participants' physical coronal plane, and negative and positive values indicate the rear and frontal spaces, respectively. The VOTs (±*SD: Standard deviation*) are 6.9 ± 5.6 s for 0.15 m/s^2^ and 6.4 ± 5.2 s for 0.3 m/s^2^ in the forward condition and 5.8 ± 4.3 s for 0.15 m/s^2^ and 5.4 ± 4.0 s for 0.3 m/s^2^ for the backward condition. For each self-motion direction, a repeated-measures analysis of variance (ANOVA) with one within-participant factor (no motion, 0.15, and 0.3 m/s^2^) was performed for the sound localization data. For the backward condition, a significant effect of experimental condition [*F*_(2, 22)_ = 6.59, *p* = 0.006] was observed. The mean sound position aligned with participants' SCP significantly shifted backward. In other words, sounds that were actually located in the traveling direction were perceived as being biased toward the null point. Multiple comparisons (Tukey's HSD, α < 0.05) revealed that the magnitude of mislocalization increased as acceleration increased. However, no effect of experimental condition was observed in the forward motion condition [*F*_(2, 22)_ = 2.58, *p* = 0.098]^2^.

In order to investigate the relationships between the strength of self-motion perception represented by the VOTs and the perceived sound positions, we calculated the correlation coefficient in each condition. However, no significant correlations were observed (*r* = 0.36, *p* = 0.25 for 0.15 m/s^2^ and *r* = 0.08, *p* = 0.81 for 0.3 m/s^2^ in the forward condition and *r* = −0.32, *p* = 0.32 for 0.15 m/s^2^ and *r* = −0.37, *p* = 0.24 for 0.3 m/s^2^ in the backward condition).

## Experiment 2

Experiment 1 demonstrated that visual linear acceleration information influenced the perceived sound position aligned with the SCP. Participants perceived sound sources that were physically located ahead of them in the traveling direction as aligned with their SCP. This is consistent with our previous study using vestibular stimulation. However, there is a critical difference: while the effect was only observed in the forward self-motion condition in our previous study, it was mainly observed in the backward self-motion condition in Experiment 1 of the present study. One critical difference between the visual and vestibular self-motion processing systems is that the visual system can detect self-motion at constant velocity, while the vestibular system cannot. Most studies investigating the visual illusion of self-motion have used constant velocity to minimize vestibular-visual conflict as much as possible (“visual-vestibular conflict theory,” Zacharias and Young, 1981) and to maximize the effect of visual stimulation. Thus, such assumed visual-vestibular conflicts (i.e., relatively weak self-motion perception) might somehow influence the integration of self-motion information into auditory space representations. Experiment 2 tested this possibility by using visual stimulation with a constant velocity, instead of acceleration.

### Methods

Nine participants (all males; age range: 21–40 years), including two of the authors, took part in this experiment. Three of them (two authors and one volunteer) also participated in Experiment 1. All participants other than the authors were naïve to the purpose of the experiment. All participants had normal or corrected-to-normal vision, normal hearing, and no vestibular dysfunction. Informed consent was obtained from each participant before the experiment.

The experimental setup and stimuli were the same as those used in Experiment 1, with the following exceptions. The random-dot pattern displayed on the screen simulated either forward or backward linear self-motion at a constant velocity (1.5 m/s). The no motion condition was also included, but no dot pattern was presented, different from Experiment 1. This was to eliminate a possibility that the static dot pattern had some influence on sound localization (i.e., judgment bias found in the no motion condition in Experiment 1). Each participant completed one experimental block, in which these three conditions (forward, backward, and no motion) were presented in a randomized order. In the forward and backward conditions, the static random-dot pattern was presented with a fixation point at the beginning of each trial. When the participants pressed a button of the gamepad, the pattern started to move. Two seconds after the participants reported self-motion perception, a target sound was presented. The time between participants' button press and target sound presentation was shorter than in Experiment 1. However, we confirmed that the strength of self-motion perception was nearly unchanged in our experimental setup even if the random-dot pattern was presented for a longer period of time in the preliminary experiment. In the no motion condition, only a fixation point was presented at the beginning of each trial. Two seconds after the participants press a button of the gamepad, a target sound was presented.

### Results and discussion

**Figure 4:**
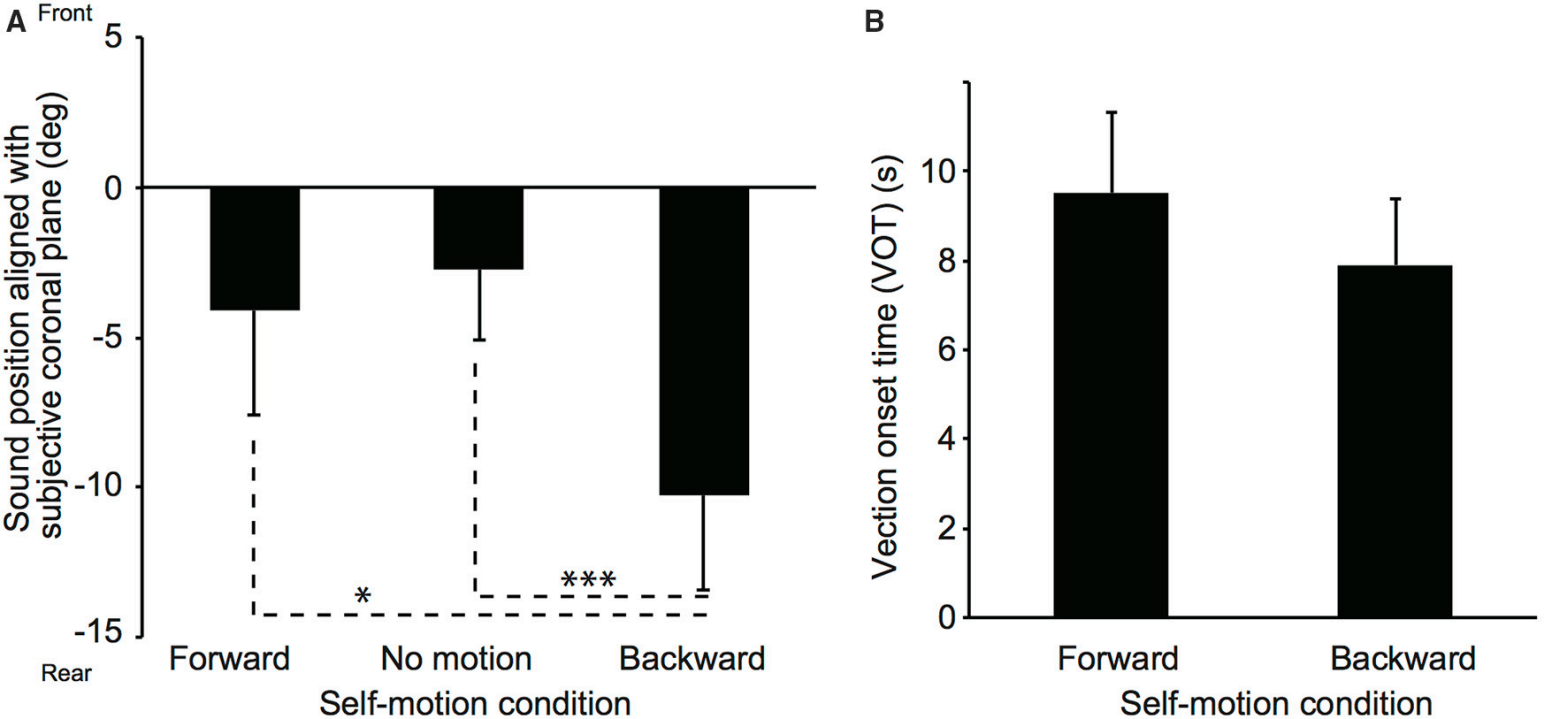
**Effect of constant velocity on auditory localization (A) and on vection onset latency (B) in Experiment 2**. The simulated self-motion velocity was 1.5 m/s. Error bars denote standard errors. Note that the participants experienced self-motion perception but were not physically moved. ^*^p < 0.05; ^***^p < 0.005.

Two participants were excluded from the data analysis because of low sound localization accuracy (e.g., front-back confusion) in the practice block. Figure 4 shows mean sound positions aligned with participants' SCP and mean VOTs in Experiment 2. The null point indicates a sound position aligned with participants' physical coronal plane, and negative and positive values indicate rear and frontal spaces, respectively. The VOTs are 9.5 ± 4.8 s and 7.9 ± 4.0 s for the forward and backward conditions, respectively. A repeated-measures analysis of variance (ANOVA) with one within-participant factor for the sound localization data revealed a significant effect of experimental condition [*F*_(2, 12)_ = 6.94, *p* = 0.010]. Multiple comparisons (Tukey's HSD, α < 0.05) revealed that mean sound position aligned with participants' SCP was significantly displaced backward in the backward compared with no motion and forward conditions. No significant difference was observed between the no motion and forward conditions. Thus, these results suggest that the effect of visual self-motion information on sound localization in depth was salient for the backward self-motion condition, regardless of motion type (acceleration or constant velocity)^3^. In order to investigate the relationships between the strength of self-motion perception represented by the VOTs and the perceived sound positions, we calculated the correlation coefficient in each condition. However, no significant correlations were observed (*r* = 0.14, *p* = 0.76 and *r* = −0.04, *p* = 0.93 for the forward and backward conditions, respectively).

In both Experiments 1 and 2, there were slight shifts of sound positions aligned with the SCP in the backward direction even in the no motion condition. In general, front-back discrimination of sound sources is more difficult than those for left-right discrimination, because simple interaural cues such as interaural intensity and time differences cannot provide useful information (Blauert, 1983). Thus, one may argue that the observed shifts have something to do with the intrinsic characteristics of the auditory system and the double staircase procedure used in the present study. However, our previous study confirmed that displacements of sound localization during self-motion were observed not only in the double-staircase procedure but also in the pointing task in which target sounds were presented only in the frontal space (Teramoto et al., 2012). Furthermore, with both procedures, the sound positions aligned with the SCP were displaced slightly backwards in the no motion condition. Thus, we think that the double-staircase procedure would have less to do with the observed shifts.

## General discussion

The present study demonstrated that the sound position aligned with the SCP was displaced in the direction of visually induced self-motion compared with a no motion condition. The sound source located ahead in the traveling direction was perceived closer to the null point. This effect was mainly observed in the backward condition irrespective of motion type (acceleration or constant velocity) and increased with an increase in acceleration.

In our present and previous studies, a number of acoustic cues were available to participants. These include not only acoustic cues for distance perception, such as intensity and HRTF parallax (Zahorik et al., 2005), but also those for azimuthal direction perception, such as interaural differences. In our previous study (Teramoto et al., 2012), these cues varied during the 30-ms target sound presentation between the self-motion vs. static conditions. In contrast, these were consistent between the static and self-motion conditions in the present study. Thus, the present study indicates that self-motion information itself is more critical for the distortion of auditory space during self-motion than the continuous change in acoustic cues, and that some integration process between auditory and self-motion information contributes to this phenomenon.

For rotary stimulation, the perceived position of a sound source shifted in the opposite direction of self-motion due to both visual (Thurlow and Kerr, 1970) and vestibular stimulation (Münsterberg and Pierce, 1894; Clark and Graybiel, 1949; Arnoult, 1950; Lester and Morant, 1970). In linear stimulation, the sound position aligned with the SCP was displaced in the direction of self-motion such that sound sources located in the traveling direction were perceived as closer to the listener than their actual positions (i.e., displacement in an opposite direction of self-motion) (Teramoto et al., 2012). Thus, the displacement direction of the sound source during visual stimulation in the present study is consistent with previous studies. Several studies have attributed auditory mislocalization during vestibular rotary and gravitoinertial force stimulation to shifts in subjective body positions or egocentric reference frames (Münsterberg and Pierce, 1894; Clark and Graybiel, 1949; Arnoult, 1950; Lester and Morant, 1970). That is, subjective body positions or egocentric reference frames are displaced in the opposite direction of self-motion so that a sound source appears to be displaced in the opposite direction of self-motion. However, our recent study suggests that auditory mislocalization during forward self-motion is likely caused by a compression of auditory space. This is because all the auditory stimuli located in the traveling direction were perceived as closer to the null point and localization error increased with increasing distance from the null point compared with the no motion condition. Given that the present study investigated the same type of self-motion (linear self-motion) and sound localization (i.e., depth), the results of the present study may reflect the same underlying mechanism as our previous study. However, it is not evident whether the current results are due to a shift in the frame of reference or compression of auditory space. This should be addressed in future studies.

It should be noted that there is a critical difference between our previous (i.e., vestibular stimulation) and present (i.e., visual stimulation) studies. While distortion of auditory space was only observed in the forward self-motion condition in our previous study, it was mainly observed in the backward self-motion condition in the present study. In Experiment 2, visual stimulation of a constant velocity was used instead of constant acceleration to reduce the assumed visual-vestibular conflicts as much as possible. However, this difference was still observed. Several studies have reported asymmetries between forward and backward self-motion. For example, visual aftereffects can be inhibited when forward (real) self-motion is combined with expanding visual flow (Wallach and Flaherty, 1975; Harris et al., 1981). This phenomenon corresponds to our ordinal experience of fewer motion aftereffects after driving a car (e.g., Addams, 1834) than after seeing a moving pattern while stationary. The vestibular system likely inhibits motion processing in the visual system. Interestingly, these aftereffects are not observed when backward self-motion is combined with contracting visual flow. This suggests that there is a stronger connection between the visual and vestibular systems during forward vs. backward self-motion. Studies on linear vection have also reported an asymmetry in vection strength between forward and backward self-motion conditions: stronger self-motion is induced by a contracting vs. expanding flow patterns (Berthoz et al., 1975; Bubuka et al., 2008). In the present study, most participants retrospectively reported stronger self-motion perception for the backward than forward self-motion condition, although there was no correlation between the VOTs and perceived sound positions. Bubuka et al. (2008) explain this asymmetry in self-motion perception with respect to an individual's exposure history. Specifically, greater experience moving forward than backward strengthens expectations about the contingency between visual and non-visual signals in the forward self-motion condition, so that larger sensory conflict arises from expanding vs. contracting flow patterns when there is no vestibular input. Furthermore, a “source separation problem” in the visual system could affect self-motion perception. The visual system has to discriminate between object motion and self-motion from retinal inputs. In particular, detecting approaching objects is essential for survival. The visual system plays an important role in this task because of its high spatial resolution. For self-motion detection, the other systems, such as the vestibular and proprioceptive systems, can help. Therefore, sensitivity to forward self-motion in the visual system might be relatively low. To compensate for this disadvantage of the visual system, the vestibular system might be more sensitive to forward vs. backward self-motion. Thus, we speculate that self-motion processes are not sufficient to induce the distortion of auditory space in the forward self-motion condition in the present study and the backward self-motion condition in our previous study. Further experiments are needed to clarify this point.

In conclusion, the present study revealed that the sound position aligned with the SCP was displaced in the direction of visually induced self-motion especially in the backward self-motion condition as compared with a no motion condition. Considering our previous data together (Teramoto et al., 2012), these results suggest that self-motion information, irrespective of its origin (the vestibular or visual systems), is crucial for auditory space perception. The underlying mechanisms for this phenomenon should be addressed in future studies.

### Conflict of interest statement

The authors declare that the research was conducted in the absence of any commercial or financial relationships that could be construed as a potential conflict of interest.

## Footnotes

^1^The present study defines auditory space as perceptual space but not physical space. Blauert (1983) described auditory events as “what is perceived auditorily,” distinguishing them clearly from physical phenomena. Based on this terminology, he defined as auditory space as the space constituted by “the totality of all possible positions of auditory events (p. 4),” and stated, “the locatedness of an auditory event is described in terms of its position and extent, as evaluated in comparison with the positions and extents of other objects of perception.” Since the present study investigated the perceived direction of sound sources relative to perceived body position, the auditory space investigated in the present study was exactly in his definition of auditory space.

^2^We conducted ANOVAs after removing the authors' data. The results of the analyses were very similar to those of the original analyses. A significant effect of experimental condition was observed for the backward condition [*F*_(2, 18)_ = 4.75, *p* = 0.022] and multiple comparisons (Tukey's HSD, α < 0.05) revealed that the magnitude of mislocalization increased as acceleration increased. In contrast, no effect was observed for the forward condition [*F*_(2, 18)_ = 1.53, *p* = 0.244].

^3^The ANOVA after removing the authors' data revealed very similar results to the original analyses. We found a significant effect of experimental condition [*F*_(2, 8)_ = 7.49, *p* = 0.015]. Multiple comparisons (Tukey's HSD, α < 0.05) revealed that the displacements in the backward condition were significantly larger than those in no motion and forward conditions.
