# Supplementary material for: Distortion of auditory space during visually induced self-motion in depth
Source: Front Psychol. 2014 Aug 5;5:848. doi: 10.3389/fpsyg.2014.00848 (PMC4122181; doi:10.3389/fpsyg.2014.00848)
Supplement: Supplementary file 1 [file Presentation1.PDF]

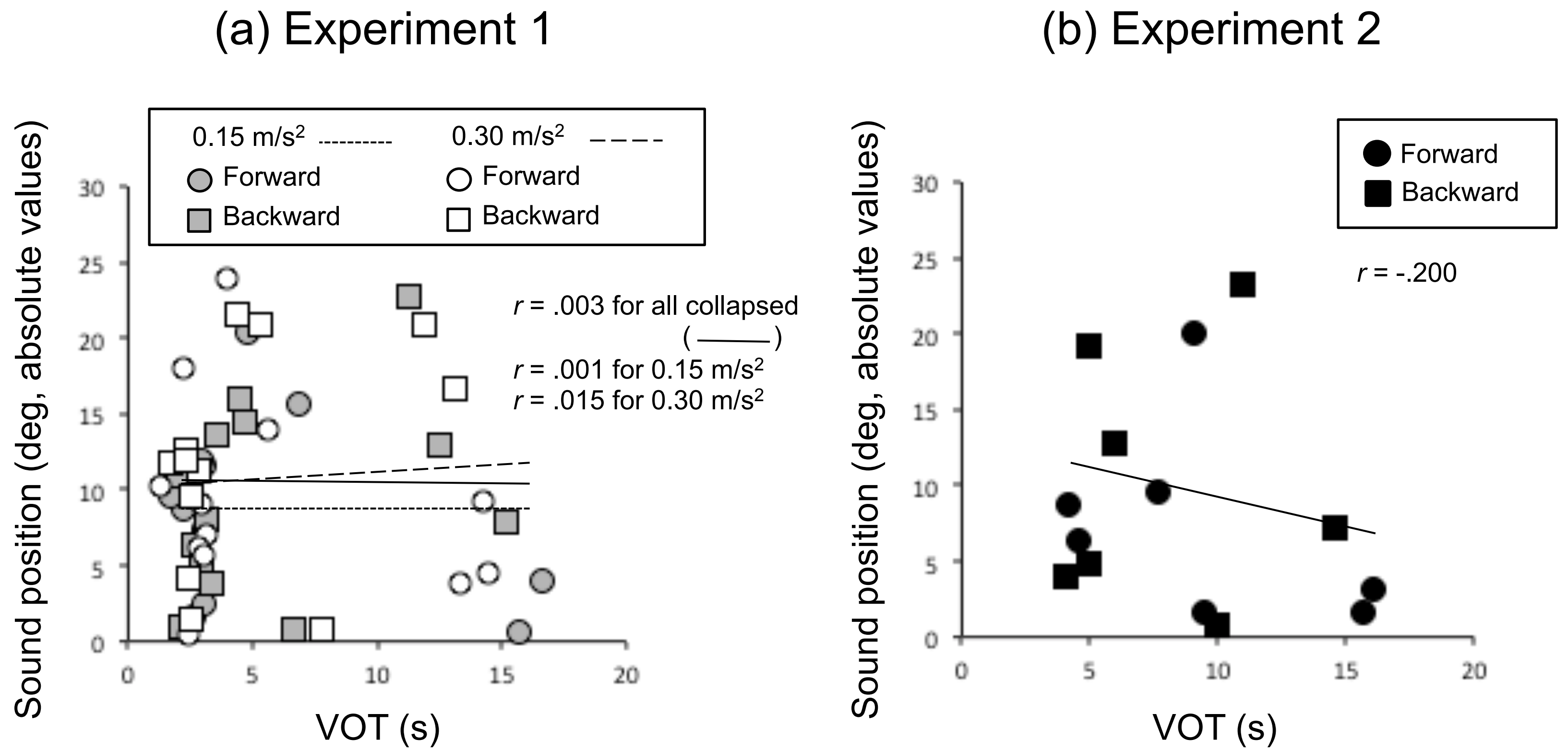

Figure S1. Relationship between VOTs and sound positions aligned with the SCP in Experiments 1 (a) and 2 (b). The localization data for the forward and backward conditions were converted into absolute values. Correlation coefficients (*rs*) were calculated after collapsing across the forward and backward conditions (the graph (a) also includes a correlation coefficient after collapsing across the acceleration conditions). Each data point represents the localization data for one participant.

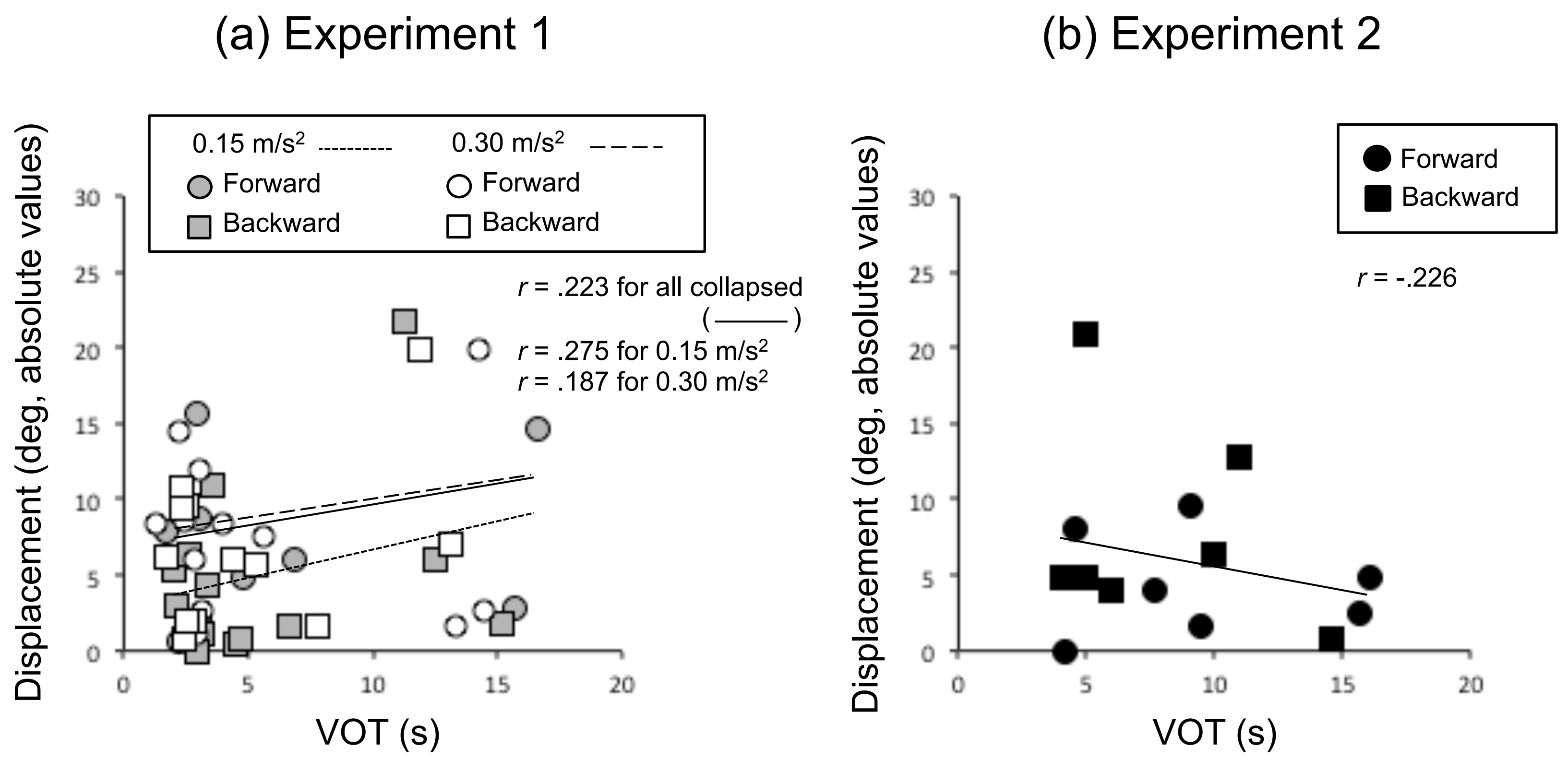

Figure S2. Relationship between VOTs and position shifts relative to the no motion condition in Experiments 1 (a) and 2 (b). The position shifts were calculated by subtracting the data for the no motion condition from those for the backward and forward conditions. The shift data were, then, converted into absolute values. Correlation coefficients (*rs*) were calculated after collapsing across the forward and backward conditions (the plot (a) also includes correlation coefficient after collapsing across the acceleration conditions). Each data point represents the position shift data for one participant.
